# Effect of Body Position on Intraocular Pressure Measured by Rebound Tonometer in Healthy Children

**DOI:** 10.4274/tjo.galenos.2020.57702

**Published:** 2020-10-30

**Authors:** Dilek Uzlu, Nurettin Akyol, Adem Türk, Nurcan Gürsoy, Ahmet Mehmet Somuncu, Yavuz Oruç

**Affiliations:** 1 Karadeniz Technical University Faculty of Medicine, Department of Ophthalmology, Trabzon, Turkey; 2 University of Health Sciences Turkey, Fethi Sekin City Hospital, Clinic of Ophthalmology, Elazığ, Turkey

**Keywords:** Child, intraocular pressure, body position, ocular tonometry

## Abstract

**Objectives:**

To evaluate the effect of body position on intraocular pressure (IOP) measurement in the pediatric age group.

**Materials and Methods:**

Children whose general condition was healthy and ophthalmic examination was within normal limits were included. Forty-nine eyes of 49 pediatric patients were included in the study. IOP was measured with an ICARE rebound tonometer (ICARE PRO; ICARE, Helsinki, Finland) while patients were in standing, sitting, and supine positions. Differences between the consecutive measurements were compared statistically.

**Results:**

Twenty-two of the 49 patients were female, 27 were male. The mean age was 9.61±2.66 (5-15) years. Mean IOP values in the standing, sitting, and supine positions were 18.81±2.97 (11.6-26.2) mmHg, 18.88±3.44, (12-28.2) mmHg, and 19.01±2.8 (13.5-25.9) mmHg, respectively. There were no statistically significant differences in pairwise comparisons of the measurements taken in the different positions (p=0.846, p=0.751, p=0.606). There was a statistically significant correlation between corneal thickness and intraocular pressure values in all measurements (p=0.001, r=0.516).

**Conclusion:**

IOP values measured with the ICARE rebound tonometer in healthy children are not affected by body position.

## Introduction

As in adult patients, the accurate measurement of intraocular pressure (IOP) plays an important role in the diagnosis, treatment, and follow-up of pediatric glaucoma. IOP is still the only modifiable risk factor for preventing glaucoma progression.^1,2^ Evaluation of IOP in pediatric patients may vary based on the patient’s age and compliance.

The rebound tonometer (RT) is a portable tonometer that does not require topical anesthesia. In previous studies it was reported that RT is easy to use and provides reliable results in the pediatric age group.^3,4^ RT measurements strongly correlate with those obtained with a Goldmann applanation tonometer (GAT).^3,4,5^ Because evaluating changes such as visual field and retinal nerve fiber losses is difficult is in the follow-up of glaucoma in pediatric patients, it is important for IOP measurements to be accurate and reliable. During examination in outpatient clinics, IOP measurements are often obtained from infants while recumbent and from children while standing or sitting. In the literature it is reported that body position affects IOP and that IOP measurements made in supine position are higher in adult patients. In previous studies, changes in IOP due to body position were found to differ by 0.2-5.9 mmHg in measurements with different tonometers.^6,7,8,9,10^ There is little information in the literature about how IOP changes with body position in children. Determining IOP changes associated with body position is important in terms of standardizing IOP measurements in the follow-up of pediatric patients.

In this study, we aimed to evaluate the relationship between IOP values measured with an RT in different body positions in healthy children.

## Materials and Methods

The study included 49 eyes of 49 healthy children under the age of 15 years who presented to the ophthalmology outpatient clinic for routine eye screening between September 2019 and December 2019. Children with no ocular disease other than refractive errors were included in the study. Exclusion criteria were high myopia or hypermetropia (>6.00 D), corneal astigmatism >2.5 D, any known ocular disease (glaucoma, ocular hypertension, uveitis, corneal pathology), history of ocular surgery, contact lens use, use of any systemic or ocular medication that might affect IOP, and non-compliance with measurements. The study was carried out in accordance with the principles of the Declaration of Helsinki and ethics committee approval was obtained. Before the study, written informed consent was obtained from the parents of the participating children. All of the children underwent a full ophthalmologic examination in which best corrected visual acuity levels and slit-lamp anterior and posterior segment examination findings were recorded in detail. Corneal thickness measurements were performed using an optical biometry device (NIDEK, AL-SCAN, Japan).

IOP measurements were performed by the same experienced researcher using an RT (ICARE PRO; ICARE, Helsinki, Finland) in the morning between 9:00 and 12:00. When the children arrived in the outpatient clinic for examination, their IOP was first measured with the ICARE RT after they remained standing for 5 minutes. They were then instructed to sit for 5 minutes, after which the second IOP measurements were obtained. Finally, after 5 minutes lying in supine position with no pillow, the third IOP measurement was performed. Measurements were made with the ICARE RT at an appropriate distance from the central cornea while the children’s eyes were open and looking straight ahead, without use of a speculum or manual intervention to open the eyelids. Children who did not open their eyelids voluntarily, squinted their eyes, tried to open their eyelids with their hands, cried during measurement, did not comply with any of the measurement positions, or did not complete all of the measurements were not included in the study. For each body position, at least 6 consecutive measurements were made and the average IOP value was recorded. The ICARE tonometer displays results in green when the variation between measurements is within normal range, yellow when at the upper limit, and red when high. Because green results are reliable, only measurements displayed in green were used for statistical analysis.

### Statistical Analysis

Numerical data were expressed as mean ± standard deviation (SD). Statistical analysis was performed using SPSS software (SPSS, version 13.0.1, Chicago, IL, USA; license: 9069728). Comparisons between the 3 consecutive measurements were made using repeated measures analysis of variance (ANOVA). Kolmogorov-Smirnov test was used to determine whether the data were normally distributed. P<0.05 was accepted as the limit of significance. The relationship between central corneal thickness (CCT) and mean IOP measurements was evaluated using Pearson’s correlation test.

## Results

A total of 49 healthy children, 22 girls (44.9%) and 27 boys (55.1%), were included in the study. The mean age of the study group was 9.61±2.66 (5-15) years. Their mean Snellen best corrected visual acuity level was 0.97±0.08 (0.7-1). Mean IOP values in the right eye were 18.81±2.97 (11.6-26.2) mmHg in standing position, 18.88±3.44 (12.0-28.2) mmHg in sitting position, and 19.01±2.80 (13.5-25.9) mmHg in supine position. There was a difference of -0.067±0.34 mmHg in IOP measurements between standing and sitting position, -0.129±0.4 mmHg between sitting and supine positions, and -0.196±0.37 mmHg between standing and supine positions. The comparison of IOP values measured in different body positions can be seen in Table 1. The mean CCT value of the eyes in which IOP was measured was 552.93±29.04 (499-606) µm.

There was a statistically significant positive correlation between the mean IOP and CCT values obtained in the 3 different positions (p=0.001, r=0.516).

## Discussion

IOP remains the only modifiable risk factor in the prevention of glaucoma progression. Therefore, it is extremely important to measure IOP accurately and reliably. Although postural changes in IOP are common, the mechanisms underlying these IOP fluctuations are not fully understood. Looking at the literature, most previous studies were conducted in adults and compared IOP measurements obtained in sitting and recumbent positions.^7,8,9,10^ In most studies, higher IOP was reported in the supine position, but in other studies IOP did not change or decreased in the supine position.^7,8,9,10,11,12,13^ IOP elevation in the recumbent position has been attributed to an increase in episcleral venous pressure.^14,15,16^ In glaucoma patients, postural IOP changes are greater in magnitude.^17,18,19,20^

Because children are not tall enough to sit at the instruments in outpatient clinics, measurements are frequently performed in the supine or standing position. The ICARE RT is a recently introduced tonometer that provides rapid, repeatable, and highly reliable IOP measurements. Although contact with the eye causes children anxiety when using the ICARE RT, its ability to obtain measurements faster than the blink reflex allows it to be used comfortably in the pediatric age group. There are few studies in the literature that examine postural changes in IOP measurements in children. Dosunmu et al.^21^ compared IOP measurements in sitting and supine position with the ICARE RT and Tonopen in pediatric patients with and without glaucoma and reported an increase in supine IOP of +0.9±2.3 mmHg with ICARE RT and +0.7±1.8 mmHg with the Tonopen. In our study, there was a difference of 0.13 mmHg between sitting and supine position, but it was not statistically significant. The smaller IOP elevation in our study compared to that observed by Dosunmu et al.^21^ may be explained by the fact that our study group consisted of only healthy pediatric patients.

In our study, no statistically significant difference was observed in IOP measurements performed in healthy pediatric patients in standing, sitting, and supine positions. The postural increase in IOP observed in the children in our study was less than that of adults. This smaller increase in the pediatric age group may be related to children’s smaller body mass and smaller episcleral venous pressure change. Sultan and Blondeau^14^ evaluated seated and recumbent episcleral venous pressures in young adults and elderly patients and reported that the elderly group had higher episcleral venous pressure compared to the younger age group.

Changes in CCT with different body positions have been demonstrated in previous studies. Maslin et al.^22^ showed that CCT decreased in the supine position in open-angle glaucoma patients and healthy subjects, with similar changes in both groups. Fogagnolo et al.^23,24^ reported that IOP and CCT exhibited diurnal variation and that IOP fluctuations in different body positions were independent of fluctuations in CCT. Because CCT measurements in different body positions were not evaluated in our study, comparisons could not be made.

One of the weaknesses of our study was the inability to measure with a GAT, which is the gold standard for IOP measurement. Because IOP measurements cannot be obtained from children in the recumbent and standing positions with the GAT, the ICARE RT was used as the measurement method. The small number of patients is another weakness of our study. In addition, we did not include the children’s blood pressure measurements or body mass index values in the study. In order to standardize the measurements made in our study, IOP measurements were performed first in standing position, then sitting, and finally in supine position. We cannot rule out the possibility that changing this order would yield different results. Our study included healthy pediatric cases; further studies are needed to examine IOP associated with different body positions in pediatric glaucoma patients, who may show larger postural IOP changes.

## Conclusion

In conclusion, in this study it was determined that IOP measurements performed with the ICARE RT in healthy children in different body positions did not vary significantly. In the pediatric age group, the ICARE RT can be used to obtain IOP measurements either in supine position under general anesthesia or in supine or standing position in outpatient clinic examinations. Further research is needed to understand the effect of postural changes on IOP in pediatric glaucoma patients.

## Figures and Tables

**Table 1:**
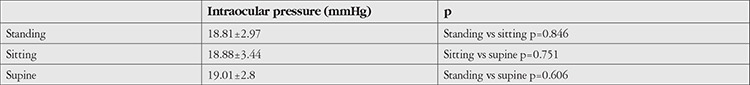
Comparison of intraocular pressure values measured in different body positions
